# Contributions to the History of Development of the Teeth

**Published:** 1888-06

**Authors:** Carl Heitzmann, C. F. W. Bödecker


### CONTRIBUTIONS TO THE HISTORY OF DEVELOPMENT OF THE TEETH.

BY CARL HEITZMANN, M. D., AND C. F. W. BÖDECKER, D. D. S., M. D. S.

CONTINUED FROM PAGE 230.

John Gibson (Der Zahnarzt, 1846, Vol. I, p. 89) gives a short description of the development of the teeth, which he concludes with the following :

I. "The human teeth are developed from the mucous membrane;

II. "The permanent teeth are in no connection with the temporary teeth, and are not products of them;

III. "The tooth germs must be regarded as onion-like formations." (Zwiebelbildungen.)

Joseph Linderer (Der Zahnarzt, 1847, Vol. II, p. 193) is of opinion that in the development of teeth a peculiar fluid is secreted in the tooth sacs, which, by coagulation, forms small round cells, out of which all other cells, canals and fibers are developed. This author observed great similarity in the structure of the dentine to that of the enamel. He describes the enamel as composed of "Enamel fibers, which consist of the peripheral fiber and a juice fiber (Saftfaser), 2 juice cells (Saftzellen), 3 cross fibers, tuft fibers (Büschel-fasern), and canals, 5 ground cells." The author also describes the lamellated appearance of the enamel, of which he mentions two kinds, the temporary, which disappears in fully formed enamel, and the permanent lamellation. The latter he locates in places at which the bundles of enamel prisms assume another direction, and are produced during the development of the enamel. On the formation of the dentine he states that the dentinal fibers are formed by the lengthening of the nucleus (Central Zelle) of the ground cell, while the basis-substance and canals are produced by the other parts of the cell. He at last declares that the cementum is formed by the dentine after the tooth has pierced the gum.

A. Krukenberg (Der Zahnarzt, 1850, Vol. V, p. 118) describes the communicating branches of the dentinal fibers, especially those in the root, while in the crown of the tooth he believes that no such communications exist, except near the enamel, where the dentinal fibers anastomose with one another by their bifurcation. He believes the dentine identical with bone in the manner of its nutrition, as he states that both the canaliculi of the dentine, and the lacunæ and their canaliculi in bone, contain a plasmatic fluid, a view which at that time was generally accepted in regard to bone tissue.

A. F. Talma (Der Zahnarzt, 1853, Vol. VII, p. 193) comes to the conclusion that the teeth, like all other parts of the animal body, must be alive.

J. E. Oudet (Der Zahnarzt, 1853, Vol. VIII, p. 257, and Vol. X, p. 65) is of opinion that teeth belong to the tegumentary formations, and have no identity with bone tissue. He also assumes that the permanent molars are derived directly from the oral mucous membrane.

Moritz Franco (Der Zahnarzt, 1856, Vol. XI, p. 1) noticed the first traces of the teeth in human embryos about the third month, but is of opinion that the teeth are not derived from the oral mucous membrane, but from the periosteum of the bone, which forms the tooth sacs, and in which the enamel organ is developed.

Natalis Guillot (Der Zahnarzt, 1858, Vol. XIII, p. 177) holds the view that the tooth germs grow from the bony portion of the jaw until they reach the mucous membrane. The germ splits up into the three different parts, out of which the three substances, enamel, dentine and cementum, are developed.

E. Mühlreiter (Deutsche Vierteljahrsschrift für Zahnheilkunde, 1868, p. 168), in an article on the arrangements of the Odontoblasts, after a careful consideration of the literature on the subject, states the results of his investigations, from which he derived the conclusion that there are two different layers of odontoblasts, one situated externally, and which, when a pulp is removed from an adult tooth, adheres firmly to the dentine. Below this layer he describes another stratum of cells which are in communication with the tissue of the pulp, to which they adhere. He was, however, unable to explain how these two layers of cells were united to each other. Regarding the formation of the basis-substance, this author is of opinion that it is formed by excretion, in which process the dentine cells are passive, but the blood-vessels of the pulp are actively engaged.

George Rollestone, in a short article read before the Odontological Society of Great Britain (Quarterly Journal of Mic. Sci. 1872, p. 109), on the Development of the Teeth of Mammals, observed that after the stellate reticulum of the enamel organ had mostly disappeared, the blood-vessels of the capsule (tooth sac) ramify closely around the enamel-forming cells.

Hohl (Deutsche Vierteljahrsschrift für Zahnheilkunde, 1869, p. 114) observed in two temporary teeth, which he extracted from the mouth of a boy seven years of age, who was suffering from hydrocephalus congenitus, that the cementum upon the roots of these teeth was very irregularly distributed. He also noticed many Haversian canals, both in the cementum and dentine, some of which were in direct communication on one side with the vessels of the pericementum, and on the other with those of the pulp. He found a great number of interglobular spaces in the dentine.

Heinrich Frey (Grundzüge der Histologie, Leipzig, 1875, p. 78) gives a short description of the development of the teeth, and only states that the dentine is formed by the odontoblasts, the enamel by the epithelial cells (ameloblasts), and the cement by the tooth-sac, but he does not mention anything of the manner in which these tissues are developed.

Joseph Hyrtl (Lehrbuch der Anatomie, Wien, 1885, p. 674) is of opinion that a primary furrow runs along the jaws, covered by epithelium, which is gradually deepened, and which, by the upward growth of tooth-sac, divides this continuous furrow into single enamel germs, one for each tooth. In regard to the formation of the enamel and dentine, he states that the enamel cells become the enamel-prisms, and the odontoblasts form the dentinal fibers around which the basis-substance is deposited.

E. Klein (Atlas of Histology, Philadelphia, 1880, p. 184) maintains that the basis-substance of the dentine is formed by the odontoblasts, while the dentinal fibers are derived from a deeper layer of the tissue wedged in between the odontoblasts, and states that the assertions of Boll may be correct, viz.: "That the non-medullated nerve fibers ascend into the dentinal canals." He further says that owing to the upward growth of the tooth-sac, the epithelial cord of the enamel organ loses its connection with the latter. He then describes the formation of the stellate reticulum from the epithelial elements in which he observed no blood-vessels. This change the author declares to be due to an accumulation of fluid between the epithelial elements, and is opposed to pronouncing it connective tissue.

Klein further states that as development proceeds and the middle membrane of the enamel organ disappears, the inner and outer membranes (epithelium) are again brought in contact, and he believes the enamel to be formed in the same manner as the dentine. He describes the transverse striæ of Retzius to be the points of union between the different enamel cells. The cuticle of the enamel (Nasmyth) this author believes to arise from the external epithelium, and the cementum to be formed from the tooth sac.

T. L. Buckingham, in a paper read before The New York Odontological Society, April 20, 1880, says: "I take exception to the theory of these two histologists (Sir J. and C. S. Tomes), that the dentinal pulp is the formative organ, and that the dentine is formed from the cells of the outer layer of the pulp. That the dentine is formed between the pulp and what is called the preformative membrane all admit, but that either of these produces it, I doubt very much. My theory is, in regard to the origin of the dentine, that either a peculiar cell is created or one is metamorphosed into a dental cell, and it multiplies in some one of the ways that cells multiply in other tissues. * * * The new cell being at first all germinal matter, soon begins to harden on the outer surface, and the most of it becomes formed material, but there always remains a portion of the germinal matter which is pushed towards the pulp and continues to grow in this direction, leaving behind it the hardened portion and a small fiber of germinal matter. With this theory a single cell would form the whole length of the dentinal tube. * * * As the space at the periphery of the dentine is greater then near the pulp, * * * crowding together takes place to a certain extent, two cells coalesce, and in this manner branches are formed.

Robert Mathes (Deutsche Vierteljahrsschrift für Zahnheilkunde, 1881, p. 252) gives a quotation from an article by Heinrich Schmidt: On rhachitic deformities of the jaws and their influence upon the teeth, in which the author (Schmidt) describes the malformations of the jaws, and also states that probably the transverse furrows upon some incisor teeth of rhachitic patients may be referred to an abnormal development of the tooth germ.

Myron D. Jewell (Dental Cosmos, Vol. XXVIII, p. 457) states that after the disappearance of the matrix (stellate reticulum) the external epithelium and the internal epithelium of the enamal organ become united into one layer of tissue, and as the stellate reticulum is not a secreting organ, it cannot furnish the lime-salts deposited in the enamel. He is, therefore, of opinion that lime-salts must be furnished through the circulation, and that the process of calcification is deferred until the outer and inner epithelium have been united into one layer, as in the former layer there are present quantities of blood-vessels. This author states that the enamel is formed at the extremities of the ameloblasts, but does not say in what way. Of the formation of dentine, he asserts that the odontoblasts recede in advance of the accumulating dentine, which odontoblasts probably are continued into the dentinal canaliculi.

(TO BE CONTINUED.)

